# The question of authorship: Whose research is it anyway?

**DOI:** 10.4103/0970-0358.63937

**Published:** 2010

**Authors:** Mukund Thatte

**Affiliations:** 402 Vimal Smruti, 770 Dr Ghanti Road, Dadar, Mumbai - 400014, India. E-mail: mthatte@vsnl.com

I write this editorial for the IJPS after a gap of two years. I had thought my days of writing editorials were over, but recent events have forced me to sit down and express my views. Over the past one year there have been three instances involving prominent and academically and clinically productive units in India, where an ex-resident or fellow has published or tried to publish (in some instances they were stopped just before the journal concerned was printed) the unit's data as his/her own work without mentioning the unit or acknowledging the contribution of several faculty in the unit. In fact, in two out of the three incidents, the person involved was not even the chief operating surgeon in the case(s) and the data may have spanned a period beyond that of the person's tenure in the unit. I am loath to mention the names of the individuals but the units concerned are PGI Chandigarh, Tata Memorial Hospital Mumbai and Ganga Hospital Coimbatore. I have the permission of all three unit heads to mention their units as the aggrieved parties.

This raises the question: Whose research is it anyway? Very often research fellows or senior residents, who are involved in data collection and/or analysis on behalf of the Chief, begin to get a feeling of ownership of the data because they have spent considerable time and energy getting it together from scattered records and patient follow ups. They typically are not the operating surgeons, have not initiated the idea/ protocol and may not even have been present when the series started in the unit. I will give you an example: Let us say Unit X started to do a particular kind of operation for a certain condition five years ago. In the 4th year of the programme, the unit head or other senior directs a resident/fellow to start collecting and collating the data because it looks promising and is worthy of publication. He or she then gathers it all together and sets up the paper 'from scratch' - thereby beginning to get a feeling of ownership of this data This is the root of the problem. It is therefore important to define authorship and ownership of data.

The website of the International Committee of Medical Journal editors defines an author:[1]

'Authorship credit should be based on: 1) substantial contributions to conception and design, acquisition of data, or analysis and interpretation of data; 2) drafting the article or revising it critically for important intellectual content; and 3) final approval of the version to be published.

Authors should meet conditions 1, 2, and 3.

The World Association of Medical Editors too has published its considered views on its website.[2]

I quote:

'Authorship is a way of making explicit both credit and responsibility for the contents of published articles. Credit and responsibility are inseparable. The guiding principle for authorship decisions is to present an honest account of what took place. Criteria for authorship apply to all intellectual products, including print and electronic publications of words, data, and images. Journals should make their own policies on authorship transparent and accessible'.

## CRITERIA FOR AUTHORSHIP

Everyone who has made substantial intellectual contributions to the study on which the article is based (for example, to the research question, design, analysis, interpretation, and written description) should be an author. It is dishonest to omit mention of someone who has participated in writing the manuscript ("ghost authorship") and unfair to omit investigators who have had important engagement with other aspects of the work.

Only an individual who has made substantial intellectual contributions should be an author. Performing technical services, translating text, identifying patients for study, supplying materials, and providing funding or administrative oversight over facilities where the work was done are not, in themselves, sufficient for authorship, although these contributions may be acknowledged in the manuscript, as described below. It is dishonest to include authors only because of their reputation, position of authority or friendship ("guest authorship").'

It is not my intention to say that fellows/residents do not qualify for being authors or co-authors. In fact, I would be the first to say that they must become co-authors in studies where they have put in a substantial amount of work. However to use a unit's data and attempt to publish it without acknowledging the unit, without informing the unit of the intention to do so (and therefore seeking the permission of the unit) and without getting the manuscript vetted by the unit for accuracy of data, is certainly dishonest and not acceptable.

There are actually three scenarios as our current Editor pointed out to me:

1. A deserving fellow/ senior resident who has put in a lot of work does not get due credit.
2. A fellow/Resident uses the unit data without their knowledge and publishes it.
3. A senior professor who has done a huge amount of work retires and is then denied access to unit data or omitted from authorship by his/her successor when the unit subsequently publishes the work.

These are all unacceptable but an Editor cannot curb these practices without an honour system being in place both systemically as well as in the minds of the authors submitting their work, the latter being crucial.

In fact, most units are very encouraging when it comes to publication and usually (there will be exceptions of unfair behavior) liberal about making a fellow/resident who is involved in the project a co-author. However, the data belong to the unit and not to individual residents who may have collated them on behalf of the chief.

IJPS will now have to make authorship criteria more explicit on its website as well as in 'Instructions to authors'. The Editor cannot be expected to be a policeman and, once these criteria are made clear and the author/s have signed a declaration that they have met them, the onus of proving their honesty will be with them and not the editor in case a dispute arises.

I rest my case and hope we do not have more such painful experiences in the future.
